# Cerebrospinal fluid levels of hypothalamic-pituitary-adrenal axis hormones in MCI and dementia due to Alzheimer’s disease: a systematic review

**DOI:** 10.1590/1980-5764-DN-2023-0031

**Published:** 2023-12-11

**Authors:** Felipe Duarte-Zambrano, Jorge A. Barrero, Ismena Mockus

**Affiliations:** 1 Universidad Nacional de Colombia, Sede Bogotá, Facultad de Medicina, Departamento de Ciencias Fisiológicas, División de Lípidos y Diabetes, Bogotá, Colombia.

**Keywords:** Alzheimer Disease, Cerebrospinal Fluid, Corticotropin-Releasing Hormone, Adrenocorticotropic Hormone, Hydrocortisone, Doença de Alzheimer, Líquido Cefalorraquidiano, Hormônio Liberador da Corticotropina, Hormônio Adrenocorticotrópico, Hidrocortisona

## Abstract

Underlying the neuropsychological manifestations of Alzheimer’s disease (AD), hypothalamic-pituitary-adrenal (HPA) axis dysregulation and subsequent hypercortisolemia have been proposed as major mechanisms driving AD progression from mild cognitive impairment (MCI) to the onset of dementia. Nonetheless, changes in cerebrospinal fluid (CSF) levels of HPA axis hormones remain controversial despite their potential in AD diagnosis and prognosis testing.

**Objective:**

This study aimed to review the evidence of the variation in CSF levels of CRH, ACTH, and cortisol in subjects with mild cognitive impairment (MCI) and AD compared with subjects without cognitive disorders.

**Methods:**

A systematic review was conducted in MEDLINE, EMBASE, and Web of Science databases on July 5, 2022.

**Results:**

Seventeen observational studies were included. The results from the compiled investigations showed that individuals with AD exhibit a significant elevation of CSF cortisol levels which appear to correlate with the presence of the ApoE-ε4 allele, being higher in those homozygous for this allele. The variation of CSF CRH and ACTH levels in AD, on the other hand, is still inconclusive. Moreover, most studies found no significant difference in CSF cortisol levels in individuals with MCI compared to healthy subjects and patients with AD.

**Conclusion:**

The findings gathered in this review disclose a significant elevation of CSF cortisol levels in AD. Future investigations are warranted to elucidate the potential use of CSF cortisol as a biomarker in AD-associated dementia.

## RESUMO

Subjacentes às manifestações neuropsicológicas da doença de Alzheimer (DA), a desregulação do eixo hipotálamo-pituitária-adrenal (HPA) e a subsequente hipercortisolemia foram propostas como mecanismos principais que conduzem a progressão da DA desde o comprometimento cognitivo leve (CCL) até o início da demência. No entanto, as alterações nos níveis do líquido cefalorraquidiano (LCR) dos hormônios do eixo HPA permanecem controversas, apesar de seu potencial no diagnóstico da DA e nos testes de prognóstico.

**Objetivo:**

Este estudo teve como objetivo revisar as evidências da variação nos níveis de CRH, ACTH e cortisol em indivíduos com comprometimento cognitivo leve (CCL) e DA em comparação com indivíduos sem distúrbios cognitivos.

**Métodos:**

Uma revisão sistemática foi realizada nas bases de dados MEDLINE, EMBASE e *Web of Science* em 5 de julho de 2022.

**Resultados:**

Dezessete estudos observacionais foram incluídos. Os resultados compilados mostraram que os indivíduos com DA apresentam uma elevação significativa dos níveis de cortisol no LCR que parecem correlacionar-se com a presença do alelo ApoE-ε4, sendo maior nos homozigotos para este alelo. A variação dos níveis de CRH e ACTH no LCR na DA, por outro lado, ainda é inconclusiva. Além disso, a maioria dos estudos não encontrou diferença significativa nos níveis de cortisol no LCR em indivíduos com CCL em comparação com indivíduos saudáveis e pacientes com DA.

**Conclusão:**

Os resultados reunidos nesta revisão revelaram uma elevação significativa dos níveis de cortisol no LCR na DA. Investigações futuras são necessárias para elucidar o uso potencial do cortisol no LCR como biomarcador na demência associada à DA.

## INTRODUCTION

The complex interplay between hypothalamic-pituitary-adrenal (HPA) axis dysregulation and the neurodegenerative course of Alzheimer’s disease (AD) suggests a neuroendocrine disturbance underlying the pathophysiology of AD-associated dementia. Psychological stress, common to both AD etiology and HPA axis hyperactivity^1^, sustains the hypothesis of neurodegeneration as a disorder associated with the allostatic load resulting from chronic exposure to stressful stimuli^2,3^ that converge in cortisol hypersecretion. Accordingly, HPA axis dysfunction in individuals with AD is evidenced by prolonged states of hypercortisolemia without disruption of adrenal circadian secretion^4^, which appear to contribute to the progression of cognitive impairment^5^. As such, the intricate relationship between HPA axis activity and AD pathogenesis exhibits a bidirectional pattern^6^; hippocampal and cortical damage associated with the formation of β-amyloid (Aβ) plaques and tau protein neurofibrillary tangles compromises the regulatory circuits of hypothalamic neuroendocrine cells^7^, and in turn, cortisol upregulation, and the consequent hyperactivation of glucocorticoid receptors promotes hippocampal neuronal degeneration and impairs synaptic plasticity^8^.

In recent years, advances in diagnostic imaging and research into molecular hallmarks of the neurodegenerative pathways concomitant to the deposition of pathogenic Aβ and tau proteins have led to a shift from the purely syndromic approach to AD^9^. The 2018 National Institute on Aging-Alzheimer’s Association (NIA-AA) Research Framework^9^ redefines AD based on the underlying pathophysiological changes documented in postmortem examinations or through *in vivo* biomarkers. AD is now recognized as a biological entity, not exclusively clinical, whose diagnosis is enhanced by biomarkers that provide a more precise characterization and understanding of the sequence of events that lead to cognitive decline^9^. Thus, the biological substrate underlying AD progression lies in the neurodegenerative mechanisms that precede the onset of symptoms by decades^10^, and their interactions with hormonal^11,12^, immunological^13,14^, and psychological^15,16^ factors impact cognitive performance and adaptability to neuronal decay^17^. Hence, the role of the HPA axis remains highly relevant as part of the complex network of biological events involved in the configuration of the pathogenic mechanisms implicated in the development of AD.

Corticotropin-releasing hormone (CRH), adrenocorticotropic hormone (ACTH), and cortisol constitute the primary effectors of the HPA axis. Given their role as neuromodulators within the limbic system^18^, these hormones are acknowledged as potential mediators of AD pathophysiology^19^. Increased serum cortisol levels have been documented as an independent biomarker of cognitive impairment progression^20^. Moreover, AD-associated hypercortisolemic states have been proposed as both a diagnostic element^21^ and a potential therapeutic target^22^ in dementia. Despite the extensive study of plasma hypercortisolism in AD, there is still inconclusive evidence regarding the changes in HPA axis hormones in cerebrospinal fluid (CSF) of subjects with AD. Quantification of these hormones in CSF, combined with measurement of Aβ^42^ and p-tau^9^, could provide a valuable complement to AD diagnosis and clinical staging. Therefore, the present study is a systematic review aiming to compile the literature on the variation in CSF levels of CRH, ACTH, and cortisol in subjects with mild cognitive impairment (MCI) and AD compared with subjects without cognitive disorders.

## METHODS

A systematic review was performed following the Preferred Reporting Items for Systematic Reviews and Meta-Analyses (PRISMA) parameters^23^ with the objective of gathering studies focused on assessing alterations in CSF levels of CRH, ACTH, and cortisol in individuals with MCI and AD-associated dementia.

### Inclusion and exclusion criteria

Eligibility criteria for study inclusion were as follows:

- investigations written in English and published in peer-reviewed journals;
- observational studies carried out in humans;
- reports of *in vivo* measurement of CRH, ACTH, and/or cortisol levels in CSF; and
- records that conducted a statistical evaluation of the difference between individuals diagnosed with AD or MCI, and subjects without cognitive impairment.

Within the included investigations, different cognitive status assessment tools and diagnostic criteria for AD and MCI were accepted. Studies were excluded in cases of:

- no comparison between healthy controls and subjects with AD or MCI;
- quantification of HPA axis hormones in fluids other than CSF;
- post-mortem investigations; and
- studies in which subjects with cognitive disorders were included without a clear definition of the etiology of dementia.

### Search strategy

For the systematic literature review, a structured search algorithm was employed using the following terms: [“Corticotropin-Releasing Hormone”], [“Adrenocorticotropic Hormone”], [“Hydrocortisone”], [“Cerebrospinal fluid”], [“Alzheimer Disease”], and [“Mild Cognitive Impairment”]. These were combined with their respective entry/emtree terms using truncators and Boolean operators (“AND” and “OR”) for the construction of the search strategy. MEDLINE (PubMed), EMBASE, and Web of Science databases were consulted on July 5^th^, 2022, and records published up to this date were extracted. The search algorithm used in each of the consulted databases is presented in Supplementary Material (https://www.demneuropsy.com.br/wp-content/uploads/2023/10/DN-2023.0031-Supplementary-Material.docx).

### Study selection

The systematic search in the databases consulted was performed by all authors. The titles and abstracts of the retrieved records were compiled and managed with the aid of Microsoft Excel 2020^®^. Following duplicates removal, two reviewers (JAB and FDZ) independently screened all titles and abstracts to exclude articles with no relevance to the objective of the review. Studies selected by title and abstract were then evaluated by full text to determine their final inclusion based on the criteria previously discussed. Disagreements in the study selection process were resolved by consensus and, when necessary, a third reviewer (IM) was consulted.

### Quality assessment

The methodological quality of the studies was assessed using the Newcastle-Ottawa Scale (NOS)^24^ to evaluate the design of nonrandomized observational studies. This scale analyzes eight items that are rated by a series of questions graded with a scoring system from 1 to 9 in three different categories: selection, comparability, and exposure/outcomes. The records included in the review are scored as follows: “high quality”: scores 7-9, “moderate quality”: scores 4-6, “low quality”: scores 0-3^24^.

### Data extraction

The following data were extracted from the included studies:

- authors and year of publication,
- characteristics of the study population,
- diagnostic criteria used to define dementia due to AD or MCI, and the scale for assessing cognitive impairment,
- results of the variation of the analyzed hormone in CSF of subjects with AD or MCI *versus* healthy controls.

Additionally, depending on the results of each study, the different associations established between CSF hormone levels, the degree of progression or severity of the pathology, and the state of cognitive function were extracted.

## RESULTS

**Figure 1.**
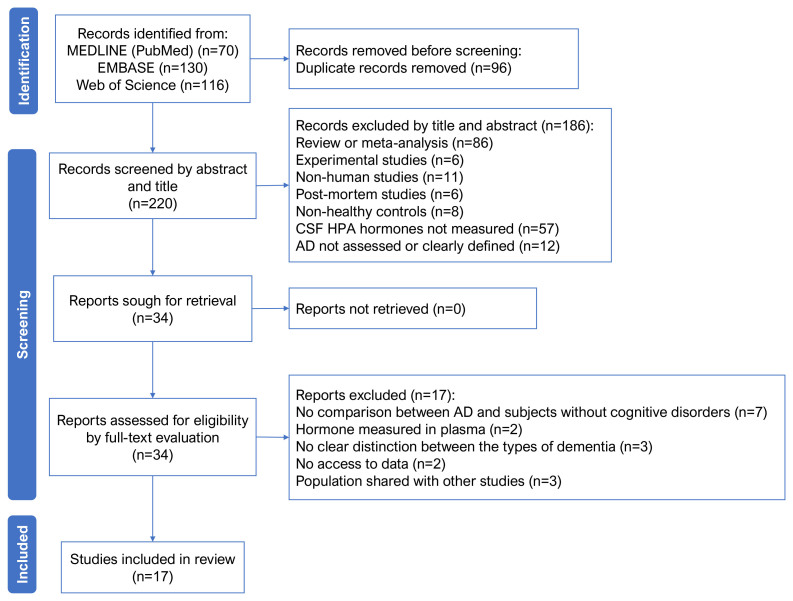
PRISMA flow diagram of studies selection.

The initial search in MEDLINE (via PubMed), EMBASE, and Web of Science databases yielded a total of 316 studies. After elimination of duplicates and screening based on title and abstract, 34 studies were obtained and underwent full-text evaluation. Finally, 17 observational studies were included (Figure 1). The characteristics of these studies are presented in Table 1 ^25-41^.

**Table 1.** Cerebrospinal fluid levels of hypothalamic-pituitary-adrenal axis hormones in mild cognitive impairment and Alzheimer’s disease.

Reference	Population	Diagnostic criteria	Hormone	Main outcomes	Quality^*^
Nappi et al.^25^	DAT group (n=20): 15 men and 5 women classified according to age at onset of AD: [1] presenile DAT [<65 years]: 14 subjects (57.3±5.5 years). Duration of disease: 2.9±1.9 years. [2] Senile DAT [≥65 years]: 6 subjects (77.5±5.9 years). Duration of disease: 3.1±2.7 years. Control group (n=12): 11 men and 1 woman (65.1±11.3 years (range 43-76 years)) under investigation for peripheral or spinal neurological diseases, with no evidence of other neurological diseases.	AD diagnosis: DSM-III criteria and exclusion of differential diagnoses. Assessment of dementia severity/cognitive status: CDR scale, MMSE, Information-Memory-Concentration test. No specific tests to assess the cognitive status of controls were described.	ACTH	CSF ACTH levels were significantly lower in subjects with DAT (4.2±3.8 fmol/mL) compared to controls (9.4±5.4 fmol/mL) (p<0.05). There was no significant difference between ACTH levels in CSF of subjects with senile DAT and presenile DAT.	High
Roelandts^26^	AD group: 17 subjects with AD. Control group: 17 subjects (age and gender matched).	AD diagnosis: 1984 NINCDS-ADRDA criteria and DSM-III criteria. No specific tests to assess the cognitive status of controls were described.	ACTH	There was no significant difference in CSF ACTH levels between subjects with AD (mean 35 pg/ml) and controls (mean 36 pg/ml) (p>0.05).	Moderate
Mouradian et al.^27^	AD group (n=16): 9 men and 7 women (63.0±1.7 years (range 53-80 years)). Subjects with moderate to severe dementia: Mattis dementia scale 105±5.7, full scale IQ 78±4.1, Wechsler memory scale 68±4. Duration of symptoms: 4.8±0.5 years (range 2-10 years). Control group (n=9): 6 men and 3 women (65.0±3.4 years (range 54 - 81 years)).	AD diagnosis: 1984 NINCDS-ADRDA criteria. Assessment of dementia severity/cognitive status: Mattis dementia scale, WAIS-R, full scale IQ, and Wechsler memory scale. No specific tests to assess the cognitive status of controls were described.	CRH	CSF CRH levels were significantly lower in AD subjects (43.0±2.5 pg/ml) *versus* control subjects (51.0±2.9 pg/ml) (p<0.05).	Moderate
Pomara et al.^28^	AD group (n=15): 7 men and 8 women (61.7±2.5 years). Subjects with moderate dementia: MMSE of 16.9±3.5, GDS of 4.7±0.5, WAIS-R of 78.7±11.7. Control group (n=10): 3 males and 7 females (63.8±3.5 years).	AD diagnosis: 1984 NINCDS-ADRDA criteria and DSM-III criteria. Assessment of dementia severity/cognitive status: MMSE, GDS, WAIS-R, and GNI scale. No specific tests to assess the cognitive status of controls were described.	CRH	There was no significant difference in CSF CRH levels between subjects with AD (65.8±5.4 pg/ml) and controls (67.1±7.1 pg/ml) (p>0.05).	Moderate
Martignoni et al.^29^	DAT group (n=16): 13 men and 3 women (65.8±8.18 years (range 56-81 years). Subjects with mild to moderate dementia: CDR scale between 1-2. Duration of symptoms: 3.5±2.8 years (range 0.5-10 years). Control group (n=21): 18 men and 3 women (65.2±0.4 years).	AD diagnosis: DSM-III criteria and exclusion of differential diagnoses. Assessment of dementia severity/cognitive status: CDR scale. No specific tests to assess the cognitive status of controls were described.	CRH	CSF CRH levels were significantly higher in subjects with AD (1.99±0.68 ng/mL (range 0.78-2.99 ng/mL)) compared to controls (0.90±0.57 ng/mL (range 0.18–2.45 ng/mL)) (p<0.01).	High
Martignoni et al.^30^	DAT group (n=10): 8 men and 2 women (mean 63.8 years (range 48-78 years). Control group (n=11): 9 men and 2 women (67.3±6.9 years (range 55-79 years) who underwent lumbar puncture for diagnostic purposes, with no evidence of neurodegenerative diseases.	AD diagnosis: DSM-III criteria and exclusion of differential diagnoses. Assessment of dementia severity/cognitive status: CDR scale. No specific tests to assess the cognitive status of controls were described.	CRH	CSF CRH levels were significantly higher in subjects with AD (462±288 pg/ml (range 156–1071 pg/ml)) compared to controls (158±122 pg/ml (range 38-470 pg/ml)) (p<0.01). CSF CRH values in AD subjects were inversely correlated with MMSE score (r=-0.53; p<0.05), but not with CDR scale score or symptom duration.	High
Molchan et al.^31^	AD group (n=49): 26 men and 23 women (66.3±8.6 years). Age of disease onset at 62.1±8.9 years. Duration of disease: 4.2±2.6 years. Control group (n=13): 10 men and 3 women (63.5±10.0 years).	AD diagnosis: DSM-III criteria and exclusion of differential diagnoses. Assessment of dementia severity/cognitive status: CDR scale. Cognitive evaluation of controls: Wechsler Memory Scale.	CRH	There was no significant difference in CSF CRH levels between subjects with AD (76.7±26.1 pg/mL) and control subjects (87.1±15.8 pg/mL) (p>0.05).	Moderate
Edvinsson et al.^32^	AD group: 39 subjects. No age reported. Control group: 11 subjects (range 23–58 years).	No diagnostic criteria for AD were specified. No specific tests to assess the cognitive status of controls were described.	CRH	There was no significant difference in CSF CRH levels in subjects with AD (9.0 pmol/L) compared to controls (15.5 pmol/L) (p>0.05).	Moderate
Heilig et al.^33^	^†^AD group (n=36): 13 men and 23 women (71.7±0.8 years (range 57–79 years)). Control group (n=40): 18 men and 22 women (79±0.8 years (range 75–85 years)).	AD diagnosis: 1984 NINCDS-ADRDA criteria. Cognitive evaluation of controls: MMSE.	CRH	CSF CRH levels were significantly lower in subjects with AD *versus* control subjects (p<0.001). CSF CRH concentration values are not reported.	Moderate
Suemaru et al.^34^	AD group: 16 subjects (79.1±4.8 years). Control group: 5 subjects (79.0±7.0 years).	No diagnostic criteria for AD were specified. Cognitive evaluation of controls: HDS.	CRH	CSF CRH levels were significantly lower in subjects with AD (43.9±4.1 pg/mL) *versus* control subjects (107.0±18.7 pg/mL) (p<0.01).	Low
Peskind et al.^35^	^†^AD group (n=64): 44 men and 20 women (67±1 years (range 35-85 years)). Of these, 7 subjects had familial AD (5 of 7 with ApoE ε3/ε3 genotype). Control group (n=34): 22 men and 12 women (71±1 years (range 61–86 years)). Of these, 11 had ε3/ε4 genotype, 17 had ε3/ε3 genotype, and 6 had ε2/ε3 genotype.	AD diagnosis: 1984 NINCDS-ADRDA criteria. Familial AD diagnosis: mutations in presenilin 1 or 2. Cognitive evaluation of controls: MMSE and CDR.	Cortisol	CSF cortisol levels were significantly higher in subjects with AD (0.82±0.03 ng/mL) compared to control subjects (0.73±0.03 ng/mL) (p<0.05). CSF cortisol concentrations varied in relation to ApoE genotype; both in control subjects (ε3/4>ε3/3>ε2/3) and in subjects with AD (ε4/4>ε3/4>ε3/3) (p<0.05).	High
Gil-Bea et al.^36^	AD group (n=27): 8 men and 19 women (68.92±9.11 years). Stable MCI group (n=26): 17 males and 9 females (61.64±10.31 years). Of these, 13 subjects had MCI with progression to AD (PMCI) (63.12±8.13 years). Control group (n=33): 14 men and 19 women with subjective cognitive impairment [complaints of cognitive impairment but no objective evidence of impairment] (57.51±6.42 years).	AD diagnosis: 1984 NINCDS-ADRDA and DSM-IV criteria. Evaluation of MCI: Criteria of the Key Symposium in Stockholm (2003). No difference between PMCI and stable MCI is stated. No specific tests to assess the cognitive status of controls were described.	Cortisol	CSF cortisol levels were significantly higher in subjects with AD compared to the control group (p<0.01). In the evaluation according to ApoE genotype, the difference in CSF cortisol was significant in ApoE ε4 carriers (p<0.05) but not in ApoE ε4 non-carriers (p=0.25). Subjects with AD and at least one ApoE-ε4 allele exhibited higher CSF cortisol levels compared to AD patients without ApoE ε4 alleles.	High
Czech et al.^37^	AD group with mild to moderate dementia [MMSE: >22] (n=53): 23 men and 30 women (69.7±69.5 years). AD group with moderate to severe dementia [MMSE: 14-22] (n=26): 12 men and 14 women (69.6±10.1 years). Control group (n=51): 24 men and 27 women (63.1±67.7 years).	AD diagnosis: 1984 NINCDS-ADRDA and DSM-IV criteria. The Hachinski ischemia scale was used to exclude vascular dementia. Assessment of dementia severity/cognitive status: MMSE. No specific tests to assess the cognitive status of controls were described.	Cortisol	CSF cortisol levels were significantly higher in subjects with mild to moderate AD (mean 6.64 ng/mL) and moderate to severe AD (mean 7.06 ng/mL) compared to control subjects (mean 5.24 ng/mL) (p<0.05). No significant difference was evident between CSF cortisol levels of patients with mild to moderate AD compared to the moderate to severe AD group.	Moderate
Popp et al.^38^	^†^AD group (n=105): 42 men and 63 women (72.95±7.42 years). ^†^MCI-AD group (n=102): 60 men and 42 women (67.35±7.92 years). ^†^Other type of MCI group (n=45): 32 men and 13 women (66.22±8.16 years). Control group (n=37): 21 men and 16 women (64.35±8.08 years) with indication for lumbar puncture for neurological conditions other than dementia.	AD diagnosis: 1984 NINCDS-ADRDA criteria. Evaluation of MCI: Key Symposium in Stockholm (2003) criteria, CERAD-NP test, TMTs A and B, and Bayer Activities of Daily Living. MCI-AD diagnosis: Memory impairment present and no evidence of cerebrovascular disease on physical examination or MRI. Diagnosis of MCI-O: Cases that did not meet MCI-AD criteria. Assessment of dementia severity/cognitive status: CDR scale. Cognitive evaluation of controls: CDR, CERAD-NP test, and TMTs A and B.	Cortisol	CSF cortisol levels were significantly higher in subjects with AD (0.555±0.387 mg/dL) and MCI-AD (0.493±0.480 mg/dL) compared to control subjects (0.252±0.251 mg/dL) (p<0.05). Subjects with MCI-AD exhibited significantly higher CSF cortisol levels *versus* subjects with MCI-O (0.239±0.218 mg/dL) (p<0.05). AD subjects exhibited significantly higher CSF cortisol levels *versus* MCI-O subjects (p<0.01). CSF cortisol levels did not differ significantly between: [1] MCI-AD and AD dementia, [2] MCI-O and control group.	High
Johansson et al.^39^	AD group (n=32): 15 men and 17 women (75 years (range 71-77 years)). Control group (n=20): 5 men and 8 women (75 years (range 70-78 years)).	AD diagnosis: 1984 NINCDS-ADRDA and DSM-IV criteria. Assessment of dementia severity/cognitive status: MMSE.	Cortisol	There was no significant difference in CSF cortisol levels between subjects with AD (6989 pg/mL (6289–7970)) and control subjects (6665 pg/mL (5705–7450)) (p>0.05).	High
Wang et al.^40^	Data extracted from the ADNI database (n=310): [1] 69 subjects with mild AD (74.9±7.6 years). [2] 149 subjects with MCI (74.8±7.2 years). [3] 92 controls (75.7±5.4 years). More than 50% of patients with MCI and AD were carriers of at least one ApoE ε4 allele.	The diagnosis of AD and MCI was assumed based on the classification of the subjects in the ADNI database. Assessment of dementia severity/cognitive status of both cases and controls: MMSE y Rey Auditory Verbal Learning Test.	Cortisol	There was no significant difference in CSF cortisol levels between subjects with AD (15.7±6.2 ng/mL), MCI (16.8±5.8 ng/mL), and controls (15.6±6.2 ng/mL) (p>0.05).	High
Wang et al.^41^	AD group (n=94): 49 men and 45 women (71.2±9.3 years (range 52–88 years)). aMCI group (n=22): 13 men and 9 women (72.3±8.2 years (range 56–84 years)). Control group (n=305): [1] <50 years (n=97): 53 men and 44 women (33.6±9.4 years (range 20–49 years)). [2] ≥50 years (n=208): 86 men and 122 women (66.7±10.0 years (range 50–100 years)).	AD diagnosis: 1984 NINCDS-ADRDA criteria. aMCI diagnosis: CDR scale and Logical Memory score. Assessment of dementia severity/cognitive status of both cases and controls: MMSE, CDR.	Cortisol	CSF cortisol levels showed a significant difference between the groups of subjects with AD, aMCI and controls; CSF cortisol concentration was significantly higher by 1.6±0.4 ng/mL in the AD group compared to control subjects (p<0.001). CSF cortisol levels in the aMCI group were higher than controls by 0.8±0.7 ng/mL and lower than those in the AD group by the same amount (0.8±0.7 ng/mL). Neither difference was significant (p>0.05).	High

Abbreviations: DAT, dementia of Alzheimer’s disease type; AD, Alzheimer’s disease; DSM-III, Diagnostic and Statistical Manual of Mental Disorders Third Edition; CDR, Clinical dementia rating scale; MMSE, Mini mental state exam; ACTH, adrenocorticotropic hormone; CSF, cerebrospinal fluid; NINCDS-ADRDA, National Institute of Neurological and Communicative Disorders and Stroke-Alzheimer’s disease; WAIS-R, Wechsler adult intelligence scale-revised; CRH, corticotropin-releasing hormone; GDS, Global deterioration scale; GNI, Global neuropsychological impairment scale; HDS, Hasegawa Dementia Scale; ApoE, apolipoprotein E gene; MCI, mild cognitive impairment; PMCI, mild cognitive impairment with progression to Alzheimer’s disease; DSM-IV, Diagnostic and Statistical Manual of Mental Disorders Fourth Edition; MCI-AD, Alzheimer’s disease type mild cognitive impairment; CERAD-NP test, consortium to establish a registry for Alzheimer’s disease test; TMTs A and B, trial making tests A and B; ADNI, Alzheimer’s disease Neuroimaging Initiative; aMCI, amnestic mild cognitive impairment. Notes: ^*^Quality assessed with the Newcastle-Ottawa scale; ^†^Significant difference in age compared with controls.

Eight studies evaluated the variation of CRH in CSF; three observed significantly lower levels in subjects with AD^27,33,34^, two found higher concentrations in individuals with AD^29,30^, and three found no significant difference compared to controls^28,31,32^. Only two studies addressed CSF ACTH variation evidencing both significantly lower levels in subjects with AD^25^ and no significant difference with controls^26^. Regarding the changes in CSF cortisol levels, five studies found that individuals with AD exhibited significantly higher concentrations^35-38,41^, whereas in two investigations there was no difference compared to subjects without cognitive impairment^39,40^. The variation in CSF cortisol levels in individuals with MCI was evaluated in four studies, out of which only one found significantly higher cortisol levels in subjects with MCI when compared to controls, but not in subjects from the AD group^38^. The remaining three studies found no significant difference in CSF cortisol levels between subjects with MCI, individuals with AD, and controls^36,40,41^. Notably, out of the seventeen included studies, only seven reported an assessment of the cognitive status of controls. None of these evaluated the variation of ACTH in CSF. The results of these investigations show both decrease^33^ and absence of significant changes in CRH^31^. In turn, three studies observed a significant increase in CSF cortisol levels in subjects with AD *versus* controls and individuals with MCI^35,38,41^, whereas two reported no significant difference^39,40^. Thus, while comprising a lower proportion, the results of these seven studies resemble the findings evidenced when analyzing the overall observations of the included investigations.

In addition to CSF hormones quantification, HPA axis activity in subjects with AD was evaluated through a stimulation test with intravenous administration of 100 μg of recombinant human CRH (rhCRH) in parallel to the measurement of plasma cortisol at 15, 30, 60, and 120 minutes. In response to HPA axis stimulation, subjects with AD showed a plasma cortisol peak at 30 min, while in controls it occurred at 60 min after rhCRH injection^29,30^. Furthermore, evaluation of HPA axis suppression response with 1 mg of dexamethasone in subjects with AD showed higher morning cortisol levels (8:00 a.m.) and lower suppression compared to the control group^30^. Notably, a direct correlation between serum and CSF cortisol concentrations was observed^35^, although evidence is uncertain as shown by the absence of a significant difference between plasma cortisol levels yet with a significant difference in CSF cortisol levels when comparing subjects with AD and controls^38^. R garding sample collection, twelve studies reported the time of lumbar puncture performed between 8:00-9:00 a.m.^25-32^ and 9:00-11:00a.m.^35,38,41^. Lastly, two investigations assessed the magnitude of CSF cortisol change in subjects with AD and MCI in relation to the presence of apolipoprotein E (ApoE) gene alleles; ApoE-ε2, ApoE-ε3, and ApoE-ε4. The results from these studies disclose that CSF cortisol levels vary according to ApoE genotype such that homozygous carriers of the ApoE-ε4 allele exhibit higher CSF cortisol levels compared with AD heterozygous individuals and those with no ApoE-ε4 alleles^35,36^.

The assessment of cognitive function was heterogeneously conducted within the included investigations. The cognitive status of subjects with AD was determined in twelve studies^25,27-31,34,37-41^ by means of at least one of the following scales: Mini-Mental State Examination (MMSE), Clinical Dementia Rating (CDR) scale, Mattis dementia scale, Wechsler Adult Intelligence Scale-Revised (WAIS-R), full scale IQ, Wechsler memory scale, Global Neuropsychological Impairment (GNI) scale, Hasegawa Dementia Scale (HDS), and Global Deterioration Scale (GDS). The severity of AD dementia was reported in four investigations with individuals with mild to moderate^29,37^, moderate^28^, and moderate to severe^27,37^ dementia. MCI was defined in four studies as amnestic MCI (aMCI)^41^, AD-type MCI (AD-MCI)^38^, MCI with progression to AD (PMCI)^36^, and classic MCI^36,40^ based on criteria of the 2003 Key Symposium of MCI in Stockholm^42^, Rey Auditory Verbal Learning Test, CDR, and/or Logical Memory score. Among the diagnostic criteria applied for AD clinical evaluation, five were based exclusively on the 1984 National Institute of Neurological and Communicative Disorders and Stroke-Alzheimer’s Disease and Related Disorders Association (NINCDS-ADRDA) cirteria^27,33,35,38,41^, five complemented these with the Diagnostic and Statistical Manual of Mental Disorders Third Edition (DSM-III) or Fourth Edition (DSM-IV) criteria^26,28,36,37,39^, and four used DSM-III or DSM-IV criteria exclusively^25,29-31^. Two studies did not explicitly state the criteria used for the evaluation of subjects with AD^32,34^, and one assumed the diagnosis from Alzheimer’s disease Neuroimaging Initiative (ADNI) database reports^40^. The distinction of AD subtypes when assessing CSF hormone levels was reported in four studies as follows: senile AD (≥65 years) and presenile AD (<65 years)^25^, familial AD, and sporadic AD^35^, and severe, moderate, and mild AD^37^.

At last, methodological quality and risk of bias were assessed by NOS, with an average score of 6.17 points. Of the seventeen included studies, nine were classified as high quality and low risk of bias^25,29,30,35,36,38-41^ with an average score of 7.22. Within the remaining studies, seven were classified as moderate quality research^26-28,31-33,37^ with an average of 5.43 points, and one study^34^ was designated as low quality research and high risk of bias with a score of 2.0.

## DISCUSSION

**Figure 2.**
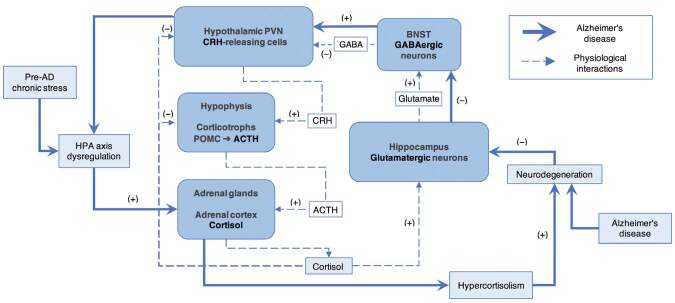
Proposed scheme of interactions between Alzheimer’s disease pathophysiology and hypothalamic-pituitary-adrenal axis dysregulation. Neuroendocrine modulation of the hypothalamic-pituitary-adrenal axis relies on the glutamatergic efferents that arise from the hippocampus to subcortical structures, mainly to neurons from the bed nucleus of the stria terminalis that convey GABAergic projections to the paraventricular hypothalamic nucleus and subsequently decrease corticotropin-releasing hormone release, thus, establishing a central negative feedback pathway through the glucocorticoidmediated increase of hippocampal excitatory inputs to the bed nucleus of the stria terminalis^51,52^. Hippocampal neurodegeneration in Alzheimer’s disease^53-55^ conditions hypothalamic-pituitary-adrenal axis hyperactivity by downregulating the negative feedback from the limbic forebrain as it has also been demonstrated in previously healthy animal models with induced Alzheimer’s disease neuropathology through Aβ intracerebral injection^56-58^.

Abbreviations: AD, Alzheimers’ disease; HPA axis, Hypothalamic-pituitary-adrenal axis; PVN, Paraventricular hypothalamic nucleus; CRH, Corticotropin-releasing hormone; BNST, Bed nucleus of the stria terminalis; POMC, Proopiomelanocortin; ACTH, Adrenocorticotropic hormone. Notes: (+) Indicates a stimulatory signal; (-) Indicates an inhibitory signal.

The findings gathered in this review support a significant elevation of CSF cortisol in subjects with AD in line with the observations of HPA axis dysfunction and elevated plasma cortisol levels documented in observational studies in humans^5,43^ and murine models^44,45^. Adrenal hyperactivity along the AD continuum suggests a neuronal exposure to elevated glucocorticoid concentrations, increasing nervous tissue susceptibility to damage associated with neurodegeneration^46^ and excitotoxicity^47^, and contributing to the progression of the canonical pathophysiological cascade of events characterizing AD^48-50^. Thus, the interactive mechanisms between AD neuropathology and HPA axis dysregulation establish a bidirectional phenomenon in which neurodegenerative involvement promotes HPA axis dysfunction and, reciprocally, HPA axis dysfunction drives neurodegeneration progression (Figure 2)^51-58^.

### Neurodegeneration and hypothalamic-pituitary-adrenal axis dysregulation

During the preclinical phase of AD, characterized by the presence of biomarkers indicative of the pathophysiological cascade preceding clinical manifestations by decades^17^, it has been shown that significantly elevated serum cortisol levels are associated with a decrease in hippocampal volume, neurogenesis, and plasticity^59^ as well as with a more accelerated decline in cognitive functions^60,61^. As central nervous system involvement progresses, a gradual loss of neuronal functionality takes place hampering the capacity to maintain clinically acceptable cognitive performance and triggering the clinical course of the disease; either mild cognitive impairment (MCI-AD) when functional independence is preserved with at least one impaired cognitive domain^62,63^, or dementia when independence is compromised due to cognitive decline in one or more domains^64^. On the other hand, during the clinical phase, disruption of the HPA axis activity is associated with the severity of cognitive impairment^65,66^, neuropsychiatric manifestations^67,68^, and greater progression of neurodegeneration with concomitant decreases in insula and amygdala volume^66^.

As evidenced by the abnormal adrenal response in AD, an attenuation of the HPA axis feedback mechanisms could take place in the context of AD, similar to the neuroendocrine disruption likely accounting for the altered CRH, ACTH, and cortisol levels in neuropsychiatric disorders^69^. While the exact mechanisms remain unknown, it has been documented that disease duration influences the degree of insensitivity to HPA axis suppression^70-72^, suggesting a gradual dissociation between the inhibitory peripheral stimulus of cortisol and the modulation of CRH secretion in the hypothalamic paraventricular nucleus (PVN) neuroendocrine cells^73^. Accordingly, cortisol secretion in response to different types of stress is altered in chronic multisystemic autoimmune^74,75^ and metabolic diseases^76,77^. From a molecular approach, several events underlie HPA axis dysregulation in states of disease which comprise an imbalance of glucocorticoid and mineralocorticoid receptors in the limbic system^78,79^, alterations of GABAergic signals modulating CRH-secreting neurons^80^, and decreased hypophyseal conversion of proopiomelanocortin (POMC) to ACTH^81^. In addition to this, neurodegeneration in AD involves a dysregulation of the HPA axis that extends beyond peripheral feedback systems compromise^82^, as studies have shown that hippocampal lesions favor a direct activation of the HPA axis^83-85^.

Studies by Martignoni et al.^30^ and Martignoni et al.^29^ showed that following intravenous administration of rh-CRH, subjects with AD reached peak plasma cortisol levels faster than subjects without cognitive impairment, but the net cortisol elevation was similar in both groups. In turn, an inverse correlation was found between age and peak plasma cortisol levels after rhCRH injection^30^. On the other hand, Molchan et al.^31^ observed that serum cortisol suppression following dexamethasone administration was significantly greater in subjects with AD than in subjects with depression. Despite the well-documented alteration in the HPA axis activity pattern in AD, the results of these studies raise uncertainty regarding the possibility that this change is a consequence of cortical degeneration, or neuropsychological alterations concomitant to the central nervous system senescence, affecting HPA axis function, as has been documented in psychiatric disorders and in aging^86^, the latter being the major risk factor for AD progression^87,88^.

### Cerebrospinal fluid cortisol in Alzheimer’s Disease

To date, literature addressing the variation of HPA axis hormones in CSF is limited. Recently, Panigrahi et al.^89^ elucidated a diurnal pattern of CSF cortisol showing that the maximum peak concentration is reached approximately two hours after the plasma peak in healthy individuals. It is now recognized that the circadian rhythm of cortisol secretion is subject to the pattern of ACTH release, which exhibits an increase in pulse amplitude between 5:00 and 9:00 a.m.^90^, resulting in morning cortisol elevation. Thus, based on the two-hour difference in maximum cortisol elevation^89^ and the absence of alteration in the circadian rhythm of the HPA axis in individuals with AD^4^, it is inferred that CSF cortisol quantification within the interval reported in the included studies (8:00-11:00 a.m.) reflects the peak levels achieved in subjects with AD.

In recent years the ApoE-ε4 allele has been recognized as a regulatory factor in cortisol elevation^91^ in addition to its direct relationship with the development of AD^92^. Apolipoprotein E modulates basal steroidogenic activity^93^ in response to ACTH stimulation in murine adrenal cortex cells^94^. Peskind et al.^35^ found that elevated CSF cortisol was associated with a higher frequency of the ApoE-ε4 allele in subjects with AD and stated that the effect of the ApoE-ε4 genotype on HPA axis activity is related to the increased risk of developing AD in carrier individuals. In addition, Gil-Bea et al.^36^ showed that the difference in CSF cortisol compared to controls was significant only in subjects with AD carrying the ApoE-ε4 allele. Among the included studies, only two evaluated the presence of the ApoE-ε4 in the studied individuals, which questions the feasibility of extrapolating the results of other investigations to the total population with AD. Thus, the findings of Peskind et al.^35^ and Gil-Bea et al.^36^ suggest the need for *ApoE* genotyping in order to establish with greater certainty its relationship with CSF cortisol variation.

In CSF, cortisol concentrations differ from plasma depending on whether the total or free fraction is measured, as free plasma levels constitute 10% of total serum cortisol^95^ whereas in CSF about 88% is not bound to binding proteins^96^. A wide variation of cortisol binding globulin (CBG) in CSF has been described with levels fluctuating between 0.3–66.0% of serum concentration^96,97^, ultimately affecting free cortisol quantification in CSF. Overall, CSF cortisol seems to be equivalent to approximately one third of the free fraction in plasma^96^. According to the results of the present review, there is a significant increase in CSF cortisol levels in AD dementia. Elevated glucocorticoids in the central nervous system have been associated with augmented tau pathology and neurodegeneration^66^. Moreover, increased cortisol levels in CSF could predict a more rapid decline of the disease^98^. While it has been described that HPA axis dysregulation occurs in the early stages of AD, accelerating the progression of dementia^38^, most studies evidenced no significant difference in CSF cortisol levels in subjects with MCI when compared to individuals with AD and healthy individuals.

### Corticotropin-releasing hormone and adrenocorticotropic hormone variation in cerebrospinal fluid

As evidenced by the records gathered in this review, older studies focused on the variation of ACTH and CRH while the most recent ones addressed cortisol changes in CSF. This framework shift might be related to the discovery of peptidergic neurotransmission systems in cognitive and behavioral functions around the 1980s^99,100^, followed later on by the uncovering of limbic-hypothalamic-pituitary-adrenal axis dysfunction in neuropsychiatric disorders^101^, thereby directing attention toward the study of cortisol alterations in the context of cognitive impairment and dementia. CRH exhibits a distinct pattern of CSF cortisol variation. It has been documented that the maximum concentration peaks are reached between 6:00 and 11:00 p.m., while the lowest levels are found around 7:00–8:00 a.m.^102^. On the other hand, in postmortem brain tissue from subjects with AD, Behan et al.^103^ identified the presence of CRH-binding protein (CRHBP) as a factor involved in the decrease of free CRH in individuals with AD and demonstrated that the dissociation of this hormone from CRHBP results in an increase in CRH levels to the values reported in control subjects. In the human brain, CRHBP is anchored to the cell membrane of neurons present in the cerebral cortex and subcortical limbic structures, and its binding function suggests a role in disease states associated with a decrease in CRH^104^. The inverse variation of CSF cortisol and CRH has been previously documented in neuropsychiatric diseases^105,106^, however, according to the results compiled in this review, the change in CSF CRH values in AD dementia remains unclear. CRHBP, by influencing circulating levels of this hormone, could represent an understudied factor underlying CSF CRH variation in AD.

Changes of ACTH in CSF have been explored to a lesser extent. The role of this hormone in different cognitive functions is well known^107^, however, exogenous administration of ACTH has not been associated with an improvement of cognitive status or a decrease in biomarkers related to altered neurotransmitter pathways in AD^108,109^. Tsigos et al.^110^ found that the presence of uncleaved POMC to ACTH in CSF can result in a positive reading of ACTH levels and should always be considered when interpreting values obtained by immunoassay techniques. CSF POMC variation is related to adiposity^111^, and the absence of a change in POMC concentration following dexamethasone administration^110^ represents a major limitation for CSF ACTH quantification. Even though there appears to be no difference in ACTH concentration in subjects with MCI and AD compared with controls^112^, the pattern of this hormone in the CSF of subjects with AD is still inconclusive and future investigations are required considering variations in measuring techniques and the presence of metabolic comorbidities in the study subjects.

### Limitations

The changing clinical approach to AD throughout history, as well as the short time window in which molecular advances in the pathophysiology and diagnosis of AD dementia have been published, pose considerable limitations to the present review. Firstly, among the included studies there is high heterogeneity in the criteria used for the diagnosis of AD and MCI, as well as in their classification into different subtypes (senile AD, presenile AD, familial AD, sporadic AD, aMCI, classic MCI, AD-type MCI), which limits the comparison of results between studies. Moreover, a sizeable proportion of studies included in this review did not assess the cognitive status of controls, so a cautious analysis of the results must be carried out, given the high prevalence of cognitive complaints in older adults. Likewise, there are several factors not reported in all studies that could affect the concentration values of HPA axis hormones in the CSF of subjects with AD, including the ApoE genotype, the age of disease onset and its duration, the severity of dementia, and the sensitivity of laboratory techniques for quantifying of hormones in CSF.

In conclusion, AD diagnosis is currently dictated by a clinical approach based on the presence of symptoms and features of cognitive impairment which could be complemented with diagnostic biomarkers that reflect the molecular pathophysiological events that precede the clinical manifestations of AD. Based on the results of this study, there is a dysregulation of the HPA axis in subjects with AD dementia, as evidenced by a significant increase in CSF cortisol levels compared to subjects without cognitive impairment. This elevation seems to be directly related to ApoE- ε4 allele frequency. On the other hand, the variation of CRH and ACTH levels in CSF of subjects with AD is still inconclusive, as the gathered studies report contradictory results. Regarding MCI, most investigations indicate that at this early stage, there is no significant difference in cortisol levels between healthy subjects and individuals with AD. At last, in agreement with the 2018 NIA-AA Research Framework^9^, the importance of approaching AD continuum as a progressive biological entity with distinct clinical stages influenced by the underlying HPA axis dysfunction is highlighted. Hence, future research aimed at elucidating the utility of CSF cortisol quantification as a complementary tool in the diagnosis, staging, and prognosis of AD dementia is warranted.
